# Supplementary material for: L-Glutamate Regulates Npy via the mGluR4-Ca2+-ERK1/2 Signaling Pathway in Mandarin Fish (Siniperca chuatsi)
Source: Int J Mol Sci. 2024 Sep 18;25(18):10035. doi: 10.3390/ijms251810035 (PMC11432707; doi:10.3390/ijms251810035)
Supplement: Supplementary file 1 [file ijms-25-10035-s001.zip › ijms-3174844-supplementary.pdf]

Supplementary Table S1. Species information.

Spiecies	Accession numbers
Homo sapiens	NM_000841.4
Mus musculus	NM_001291045.2
Danio rerio	NM_001302241.1
Oryzias latipes	XM_004068826.4
Astyanax mexicanus	XM_007253293.4
Morone saxatilis	XM_035656810.1
Oreochromis niloticus	XM_013276610.3
Latimeria chalumnae	XM_005988471.2
Ictalurus punctatus	XM_017479174.3
Takifugu rubripes	XM_003963486.3
Oncorhynchus tshawytscha	XM_042324215.1
Lates calcarifer	XM_018683907.2
Ctenopharyngodon idella	XM_051896222.1
Siniperca chuatsi	XM_044210366.1
Amphiprion ocellaris	XM_023275610.3
Cyprinus carpio	XM_042758816.1
Megalobrama amblycephala	XM_048198946.1
Epinephelus moara	XM_050038398.1
Haplochromis burtoni	XM_005917945.3
